# SARS Surveillance during Emergency Public Health Response, United States, March–July 2003

**DOI:** 10.3201/eid1002.030752

**Published:** 2004-02

**Authors:** Stephanie J. Schrag, John T. Brooks, Chris Van Beneden, Umesh D. Parashar, Patricia M. Griffin, Larry J. Anderson, William J. Bellini, Robert F. Benson, Dean D. Erdman, Alexander Klimov, Thomas G. Ksiazek, Teresa C.T. Peret, Deborah F. Talkington, W. Lanier Thacker, Maria L. Tondella, Jacquelyn S. Sampson, Allen W. Hightower, Dale F. Nordenberg, Brian D. Plikaytis, Ali S. Khan, Nancy E. Rosenstein, Tracee A. Treadwell, Cynthia G. Whitney, Anthony E. Fiore, Tonji M. Durant, Joseph F. Perz, Annemarie Wasley, Daniel Feikin, Joy L. Herndon, William A. Bower, Barbara W. Kilbourn, Deborah A. Levy, Victor G. Coronado, Joanna Buffington, Clare A. Dykewicz, Rima F. Khabbaz, Mary E. Chamberland

**Affiliations:** * Centers for Disease Control and Prevention, Atlanta, Georgia, USA

**Keywords:** severe acute respiratory syndrome, United States, surveillance, incidence, SARS virus, Coronaviridae, pneumonia, travel, respiratory tract infections

## Abstract

In response to the emergence of severe acute respiratory syndrome *(*SARS), the United States established national surveillance using a sensitive case definition incorporating clinical, epidemiologic, and laboratory criteria. Of 1,460 unexplained respiratory illnesses reported by state and local health departments to the Centers for Disease Control and Prevention from March 17 to July 30, 2003, a total of 398 (27%) met clinical and epidemiologic SARS case criteria. Of these, 72 (18%) were probable cases with radiographic evidence of pneumonia. Eight (2%) were laboratory-confirmed SARS-coronavirus (SARS-CoV) infections, 206 (52%) were SARS-CoV negative, and 184 (46%) had undetermined SARS-CoV status because of missing convalescent-phase serum specimens. Thirty-one percent (124/398) of case-patients were hospitalized; none died. Travel was the most common epidemiologic link (329/398, 83%), and mainland China was the affected area most commonly visited. One case of possible household transmission was reported, and no laboratory-confirmed infections occurred among healthcare workers. Successes and limitations of this emergency surveillance can guide preparations for future outbreaks of SARS or respiratory diseases of unknown etiology.

The emergence of severe acute respiratory syndrome (SARS) presented a challenge to public health and healthcare delivery systems worldwide. The previously unknown respiratory syndrome was characterized by nonspecific clinical symptoms, was highly transmissible in some circumstances, did not respond to antimicrobial therapy, and could rapidly progress to severe respiratory distress and death. SARS appears to have originated in Guangdong Province, China; however, the global importance of this illness was not recognized initially by local health authorities. When the World Health Organization (WHO) issued a historic global alert about cases of severe atypical pneumonia on March 12, 2003, the outbreak had spread through international travel from Guangdong Province to at least Hong Kong and Hanoi, Vietnam. There was an urgent global need for diagnosis of the etiologic agent, detection and containment of probable cases, guidance on the healthcare management of patients and potentially exposed persons, identification of measures to prevent and control infections, and timely public health communications to a wide range of audiences.

On March 14, 2003, the U.S. Centers for Disease Control and Prevention (CDC) launched an emergency public health response and established national surveillance for SARS to identify case-patients in the United States and determine if domestic transmission was occurring. We describe the surveillance system established to detect SARS in the United States, focusing on its design, challenges, and modifications that occurred as the outbreak evolved, and characteristics of the case-patients identified. Such information is critical for preparing for possible future outbreaks of SARS or other emerging microbial threats with nonspecific respiratory symptoms.

## Methods

### SARS Case Definition

CDC’s initial surveillance definition for a suspect case of SARS (Table 1) was based on a definition first published by WHO (*1*). These definitions specified clinical criteria and required a potential exposure to SARS (epidemiologic link). WHO categorized all cases with x-ray or autopsy evidence of pneumonia or respiratory distress as probable, and all others meeting the case definition were classified as suspect cases. CDC initially categorized all cases as suspect, but on April 29, 2003, CDC adopted WHO’s suspect and probable classifications (*2*).

**Table 1:** Initial SARS case definition,^a^ U.S. surveillance, March 17, 2003

Clinical criteria
Respiratory illness of unknown etiology with onset since February 1, 2003, including: Temperature >38°C Findings of respiratory illness^b^
Epidemiologic link criteria
Travel within 10 days of symptom onset to area with documented or suspected community transmission of SARS^c^ OR Close contact^d^ within 10 days of symptom onset with either a person with respiratory illness who had traveled to SARS area or a person suspected to have SARS

^a^SARS, severe acute respiratory syndrome. ^b^For example, cough, shortness of breath, difficulty breathing, hypoxia, or radiographic findings of either pneumonia or acute respiratory distress syndrome; suspect cases with either radiographic evidence of pneumonia or respiratory distress syndrome or evidence of unexplained respiratory distress syndrome by autopsy are designated “probable” cases by the WHO case definition. ^c^Hong Kong Special Administrative Region and Guangdong province, Peoples’ Republic of China; Hanoi, Vietnam; and Singapore. ^d^Having cared for, having lived with, or having had direct contact with respiratory secretions or body fluids of patient suspected to have SARS.

SARS-affected areas that constituted an epidemiologic link changed throughout the outbreak, requiring continual modification of the case definition. CDC considered an area SARS-affected if evidence of documented or suspected community transmission existed. Regions were removed from the list of SARS-affected areas when CDC-issued travel alerts or advisories were discontinued, which meant that the area had reported no new cases of SARS for 30 days.

On April 29, 2003, after a new coronavirus (SARS-CoV) was identified as the etiologic agent of SARS (*3*–*6*), the case definition was changed to incorporate criteria for laboratory-confirmed illness (*7*). Laboratory criteria were refined near the end of the outbreak, resulting in the final case definition on July 18, 2003 (Tables 2 and 3); revision of the requirements for a convalescent-phase serum specimen from 21 to 28 days after illness onset was not applied retrospectively, consistent with the instructions accompanying release of this case definition. This definition also introduced an exclusion criterion for suspect or probable case-patients confirmed negative for SARS-CoV infection. In this analysis, we did not apply this exclusion criterion to allow for a complete presentation of suspect and probable cases captured and monitored by national surveillance.

**Table 2:** CDC SARS case definition, United States, as of July 31, 2003^a^

Case classification^b^
Probable case: meets the clinical criteria for severe respiratory illness of unknown etiology and epidemiologic criteria; laboratory criteria confirmed or undetermined Suspect case: meets the clinical criteria for moderate respiratory illness of unknown etiology and epidemiologic criteria; laboratory criteria confirmed or undetermined
Clinical criteria
Asymptomatic or mild respiratory illness Moderate respiratory illness: temperature >38°C^c^ and one or more clinical findings of respiratory illness (e.g., cough, shortness of breath, difficulty breathing, hypoxia) Severe respiratory illness: criteria for moderate respiratory illness with radiographic evidence of pneumonia, respiratory distress syndrome, or autopsy findings consistent with pneumonia or respiratory distress syndrome without an identifiable cause
Epidemiologic link criteria
Travel (including airport transit ) within 10 days of onset of symptoms to area with current or recently documented or suspected community transmission of SARS (Table 3) or close contact^d^ within 10 days of symptom onset with person known or suspected to have SARS
Laboratory criteria^e^
Confirmed: detection of antibody to SARS-CoV in a serum sample; detection of SARS-CoV RNA by RT-PCR confirmed by a second PCR assay by using a second aliquot of the specimen and a different set of PCR primers; or isolation of SARS-CoV Negative: absence of antibody to SARS-CoV in convalescent serum obtained >28 days after symptom onset^f^ Undetermined: laboratory testing not performed or incomplete
Exclusion criteria
Illness fully explained by alternative diagnosis^g^ Convalescent-phase serum sample (obtained >28 days after symptom onset) negative for antibody to SARS-CoV. Case reported on basis of contact with index case subsequently excluded as SARS, provided other epidemiologic exposure criteria are not present

^a^CDC, Centers for Disease Control and Prevention; SARS, severe acute respiratory syndrome; CoV, coronavirus; RT-PCR, reverse transcriptase–polymerase chain reaction.

^b^Asymptomatic SARS-CoV infection or clinical manifestations other than respiratory illness might be identified as more is learned about SARS-CoV infection.

^c^Measured documented temperature of >38°C is preferred; however, clinical judgment should be used when evaluating patients for whom temperature of >38°C has not been documented. Factors that might be considered include patient self-report of fever, use of antipyretics, presence of immunocompromising conditions or therapies, lack of access to health care, or inability to obtain a measured temperature. Reporting authorities should consider these factors when classifying patients who do not strictly meet the clinical criteria for this case definition.

^d^Close contact is defined as having cared for or lived with a person known to have SARS or having a high likelihood of direct contact with respiratory secretions or body fluids of a patient with SARS. Examples of close contact include kissing or embracing, sharing eating or drinking utensils, close conversation (<3 feet), physical examination, and any other direct physical contact. Close contact does not include activities such as walking near a person or sitting across a waiting room or office for a brief period.

^e^Assays to diagnose SARS-CoV infection include enzyme-linked immunosorbent assay, indirect fluorescent-antibody assay, and RT-PCR assays of appropriately collected clinical specimens. Absence of SARS-CoV antibody from serum obtained <28 days after illness onset,^f^ a negative PCR test, or a negative viral culture does not exclude SARS-CoV infection and is not considered a definitive laboratory result. In these instances, a convalescent-phase serum sample obtained >28 days after illness is needed to determine infection with SARS-CoV.^g^ All SARS diagnostic assays are under evaluation.

^f^Does not apply to serum samples collected before July 11, 2003. Testing results from serum samples collected before July 11, 2003 and between 22 and 28 days after symptom onset are acceptable and will not require collection of additional sample >28 days after symptom onset.

^g^Factors that may be considered in assigning alternate diagnoses include strength of epidemiologic exposure criteria for SARS, specificity of diagnostic test, and compatibility of clinical presentation and course of illness for alternative diagnosis.

**Table 3:** Travel criteria for persons with suspect or probable SARS, United States^a^

Area	First date of illness onset for inclusion as reported case^b^	Last date of illness onset for inclusion as reported case^c^	
China (Mainland)	November 1, 2002	July 13, 2003	
Hong Kong	February 1, 2003	July 11, 2003	
Hanoi, Vietnam	February 1, 2003	May 25, 2003	
Singapore	February 1, 2003	June 14, 2003	
Toronto, Canada	April 1, 2003	July 18, 2003	
Taiwan	May 1, 2003	July 25, 2003	
Beijing, China	November 1, 2002	July 21, 2003	

^a^SARS, severe acute respiratory syndrome. ^b^The World Health Organization has specified that the surveillance period for China should begin on November 1; the first recognized cases in Hong Kong, Singapore, and Hanoi (Vietnam) had onset in February 2003. The date for Toronto is linked to laboratory-confirmed case of SARS in a U.S. resident who had traveled to Toronto; the date for Taiwan is linked to the Centers for Disease Control and Prevention (CDC) travel recommendations. ^c^The last date for illness onset is 10 days (i.e., one incubation period) after removal of a CDC travel alert. The case-patient’s travel should have occurred on or before the last date the travel alert was in place.

### Inclusion Criteria

Case-patients were eligible for inclusion if they were U.S. residents and were present in the United States during some of their illness. Non-U.S. residents who became ill or in whom SARS was diagnosed while they were in the United States were monitored as patients of special interest until April 30, 2003, after which they were included in surveillance. U.S. citizens who were not present in the United States for any period of their illness were not included in surveillance.

### National Surveillance for SARS

National surveillance began on March 17, 2003, 3 days after CDC initiated its emergency response. The analysis in this report covers the period March 17 through July 30, 2003, 3 weeks after WHO declared the global outbreak over. Case definitions were distributed to state and local health departments through CDC’s Epidemic Information Exchange (Epi-X), a secure communications network for public health professionals, and through CDC’s Health Alert Network. Case definitions were also posted on a CDC Web site dedicated to SARS. A case report form was developed to collect demographic and clinical data as well as information about epidemiologic links. This form was also distributed through Epi-X and by electronic mailings by the Council of State and Territorial Epidemiologists (CSTE) to its membership. The case report form was modified as the outbreak evolved.

At the beginning of the outbreak, health departments were requested to report to CDC all respiratory illnesses that they thought should be evaluated for SARS. Although the communication chain for reporting these illnesses to health departments varied by state, all health departments relied on passive reporting from clinicians rather than actively seeking to identify potential cases. CDC hosted weekly teleconferences with state and local health departments to address developing issues related to the domestic surveillance and response. An Atlanta-based CDC team received illness reports by telephone or fax. State and local health department personnel collected data, completed case report forms, and determined case status in consultation with CDC. When a patient met the case definition, data about that person were added to a “line list,” which was updated and analyzed daily. Hospitalized case-patients were actively monitored to establish outcomes, as were persons who had pending data that could alter case status. Illnesses that failed to meet the case definition on subsequent investigation (e.g., patient’s travel history clarified) were removed from the line list. The data collection system at both the health departments and CDC was paper-based rather than electronic or online. Epidemiologic data were entered at CDC into an electronic database that was merged with laboratory data.

### Laboratory Confirmation of SARS Infection

State and local health departments were asked to collect acute- and convalescent-phase serum and stool specimens and nasopharyngeal or oropharyngeal swab samples from all case-patients. Before the cause of SARS was established, specimens were tested for a wide array of bacterial and viral pathogens at CDC. After SARS-CoV was discovered, serum specimens were tested for SARS-CoV antibodies, and respiratory and stool specimens were tested for SARS-CoV by polymerase chain reaction (PCR) (*4*). Diagnostic testing was initially centralized at CDC. Later, reagents for SARS-CoV antibody and nucleic acid testing were made available to state public health laboratories and the Laboratory Response Network (*8*). To meet U.S. Food and Drug Administration requirements for the use of nonlicensed tests in these laboratories, CDC developed informed-consent documents and informational materials that clinicians used when collecting specimens for SAR-CoV testing from their patients. Case-patients were classified as confirmed, negative, or undetermined for SARS-CoV infection (Tables 2 and 3). On July 18, 2003, the 21-day period required for convalescent-phase specimens was extended to 28 days for newly identified cases on the basis of evidence that seroconversion sometimes occurred after day 21 (*9*).

### Laboratory Testing for Other Respiratory Pathogens

During the course of the outbreak, testing for alternative causes that could fully explain patient illness was ordered at the discretion of local clinicians, and SARS was often excluded on the basis of local interpretations of test results. Many of these illnesses were never reported to CDC. Diagnostic testing for alternative agents was performed at CDC early in the outbreak. In addition, evaluation of acute respiratory specimens and paired serum specimens from suspect and probable case-patients for evidence of the following respiratory pathogens was completed after the outbreak was over: *Mycoplasma pneumoniae*, *Streptococcus pneumoniae*, *Chlamydia pneumoniae*, *C. psittaci*, *Legionella pneumophila*, influenza viruses types A and B, respiratory syncytial virus, parainfluenza viruses types 1, 2, and 3, human metapneumovirus (HMPV), and adenovirus. *M. pneumoniae* immunoglobulin (Ig) G and IgM antibodies were measured by using the REMEL *Mycoplasma pneumoniae* IgG/IgM Antibody Test System (REMEL Inc., Lenexa, KS). *S. pneumoniae* IgG antibodies to pneumococcal surface adhesin A protein (PsaA) were measured by using a PsaA-ELISA (enzyme-linked immunosorbent assay) as previously described (*10*). A rise in IgG antibody titers of twofold or more between acute- and convalescent-phase serum pairs was considered positive for a pneumococcal exposure or event. *Chlamydia* IgG and IgM antibodies were measured by using a microimmunofluorescent antibody assay (Focus Technologies, Cypress, CA). *L. pneumophila* antibodies were measured by using an indirect immunofluorescent antibody assay (*11*). Specific IgG antibodies to the respiratory viruses (excluding influenza) were measured by using an indirect enzyme immunoassay panel, following procedures previously described for HMPV (*12*). A rise in IgG antibody titers of fourfold or greater between acute- and convalescent-phase serum pairs was considered positive for recent virus infection. Serologic analysis for influenza was performed by hemagglutination-inhibition assay. All serum specimens were treated with receptor-destroying enzyme to remove nonspecific inhibitors before testing (*13*).

Specimens from some or all of the following sources were tested by PCR for evidence of bacterial or viral infection: bronchoalveolar fluid, sputum, tracheal aspirates, nasal washings, and nasal, nasopharyngeal, and oropharyngeal swab samples. All the bacterial methods used have been described previously (*11*,*14*–*16*) except the *L. pneumophila* real-time PCR assay (Appendix).

Total nucleic acid was extracted from 100 μL of specimen by using the QIAamp Virus BioRobot MDx kit (QIAGEN Inc., Valencia, CA). Reverse transcriptase (RT)–PCR assays for influenza A and B viruses; respiratory syncytial virus; human parainfluenza viruses 1, 2, and 3 (*17*); and HMPV (*12*) were performed as previously described. RT-PCR assays for adenovirus and picornavirus (inclusive of rhinovirus and enterovirus) were performed by using these same amplification conditions with primer pairs to the conserved regions of the hexon gene and the 5′-untranslated region: adenovirus [(+) 5′-CCC(AC)TT(CT)AACCACCACCG-3′; (-) 5′-ACATCCTT(GCT)C(GT) GAAGTTCCA-3′] and picornavirus [(+) 5′-GGCCCCTGAATG(CT)GGCTAA-3′; (-) 5′-GAAACACGGACACCCAAAGTA-3′]. All nucleic acid extracts were also tested by RT-PCR for the GAPDH housekeeping gene to ensure RNA integrity and absence of RT-PCR inhibitors.

## Results

**Figure 1:**
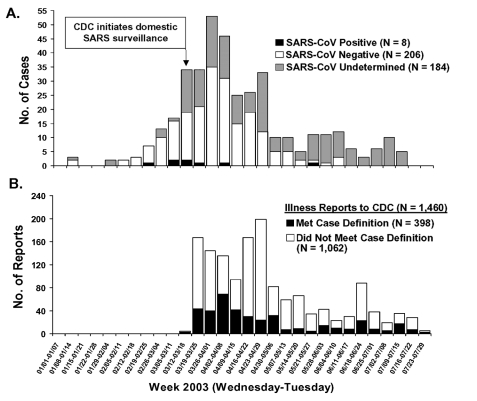
A) Number of U.S. severe acute respiratory syndrome (SARS) cases reported to Centers for Disease Control and Prevention (CDC) by week of illness onset (N = 398^a^) and B) number of unexplained respiratory illness reports received by CDC by week of illness report (N = 1,460), January–July 2003. (SARS-CoV, severe acute respiratory syndrome–associated coronavirus)

**Figure 2:**
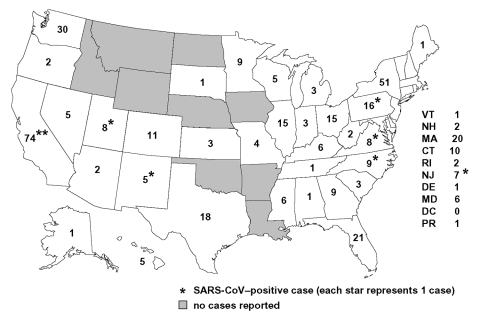
Number of suspect and probable cases of severe acute respiratory syndrome (SARS) cases reported to Centers for Disease Control and Prevention March 17–July 30, 2003, by state of residence (N = 398). (SARS-CoV, severe acute respiratory syndrome–associated coronavirus)

From March 17 to July 30, 2003, CDC received reports of 1,460 respiratory illnesses under evaluation for SARS, of which 398 (27%) met the case definition for suspect or probable SARS before laboratory-based exclusion criteria for SARS-CoV–negative status were applied (Figure 1). Seventy-two (18%) of those meeting the case definition had chest x-ray evidence of pneumonia and were classified as probable case-patients. Eight case-patients (2%) were confirmed to be positive for SARS-CoV, 206 (52%) were confirmed to be negative for SARS-CoV by serologic testing, and 184 (46%) had undetermined SARS-CoV status because of the absence of convalescent-phase serum samples. Cases were reported from 41 states and Puerto Rico, with the highest case counts in California (74), New York (51), and Washington (*30*); no cases were reported from 9 states or the District of Columbia (Figure 2).

Of the eight confirmed SARS-CoV–positive case-patients, all had radiographic evidence of pneumonia and six were identified in the first month of surveillance (Table 4). Five traveled to Hong Kong, two to Toronto, and one to Singapore. Further case details have been presented elsewhere (*18*–*21*). Among the eight confirmed SARS-CoV–positive case-patients, seven had illnesses that were associated solely with travel to an affected area. Although the eighth case-patient traveled with her spouse (subsequently confirmed as a case-patient) to an affected area (Hong Kong, where both stayed in a hotel in which intense local transmission occurred [*22*]), the epidemiologic link was classified as close contact because the onset of illness occurred 13 days after the couple’s return to the United States (*18*,*20*).

**Table 4:** Characteristics of SARS case-patients, U.S. SARS surveillance, March 17–July 18, 2003^a^

Characteristic	Overall			SARS-CoV positive		SARS-CoV negative			SARS-CoV undetermined	
	Probable, % (N = 72)	Suspect, % (N = 326)		Probable, % (N = 8)		Probable, % (N = 39)	Suspect, % (N = 167)		Probable, % (N = 25)	Suspect, % (N = 159)
Age (years)										
0–4	15	14		0		15	10		20	19
5–9	4	4		0		3	5		8	4
10–17	3	2		0		5	2		0	0 (1)
18–64	58	73		100		54	76		52	70
>65	20	7		0		23	7		20	7
*Sex*										
Female	44	47		50		41	50		48	45
Male	56	53		50		59	50		52	55
*Race*										
White	47	58		37		54	62		40	53
Black	1	2		0		0	2		4	1
Asian	40	33		63		36	28		40	38
Other	2	0 (1)		0		2	0		0	2
Unknown	10	7		0		8	8		16	6
*Exposure*										
Travel	83	81		88		87	82		84	81
Close contact	14	16		12^b^ (1)		13	17		8	14
Health care worker	0	1		0		0	0		0	1
Unknown	3	2		0		0	1		8	4
*Hospitalized*										
Yes	61	25		88		59	26		56	23
No	39	75		12 (1)		41	73		44	75
Unknown	0	1 (3)		0		0	1		0	2
Mechanically ventilated										
Yes	3 (2)	1 (2)		12 (1)		0	1 (1)		4 (1)	1 (1)
No	89	93		88		97	95		80	91
Unknown	8	6		0		3	4		16	8

^a^SARS-CoV, severe acute respiratory syndrome–associated coronavirus. ^b^This case-patient also traveled to Hong Kong and stayed at Hotel M; however, onset of illness was 13 days after returning to the United States.

The median age of all suspect and probable case-patients was 39 years (range 3 months to 91 years), and 53% were male (Table 4). Almost one third (124/398, 31%) of the patients were hospitalized. The median length of hospitalization for the 90 persons with adequate hospitalization duration data was 3 days (range 1–14). Twenty-one percent of hospitalized patients (19/91 patients with data on intensive care unit admissions) were admitted to an intensive care unit; only 2 of the 8 SARS-CoV–positive case-patients were admitted to intensive care units. Among all 398 suspect and probable case-patients, 4 (1%) required mechanical ventilation, one of whom was SARS-CoV positive (Table 4). No deaths were reported.

**Figure 3:**
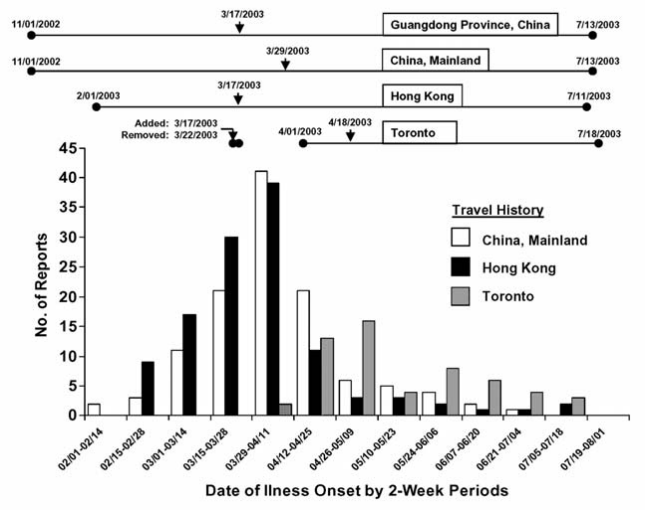
Number of suspect and probable cases reporting travel within the past 10 days to mainland China, Hong Kong, and Toronto, by date of illness onset (N = 307). Lines between solid circles denote periods during which onset of illness within 10 days of travel to the area fulfilled epidemiologic criteria for inclusion as a case of severe acute respiratory syndrome (SARS). Arrows denote the date on which an area was added to the U.S. surveillance case definition as SARS-affected.

Travel to an affected area was the most commonly reported epidemiologic link (83% of cases). Mainland China was the most frequent destination (39% of travelers), followed by Hong Kong (38%), and Toronto (18%); 22% of case-patients traveled to more than one affected area. The frequency of travel to China, Hong Kong, and Toronto among SARS case-patients is shown by date of illness onset in Figure 3; the periods during which these areas were considered SARS-affected for surveillance purposes are also shown.

No healthcare workers with suspect or probable SARS (n = 31) were confirmed to be SARS-CoV positive; 17 (55%) were confirmed SARS-CoV negative, and the remainder had undetermined SARS-CoV status. The only possible case of recognized secondary transmission was between the married couple described above.

### Number of Illnesses Reported and Completeness of Surveillance Data

The number of illnesses reported was highest during the first 6 weeks of surveillance and varied over the course of the outbreak (Figure 1). Among suspect and probable cases, the completeness of critical surveillance variables related to case definition and severity of illness was as follows: date of symptom onset, 98%; radiologic chest imaging for pneumonia, 80%; hospitalization status, 99%; hospital discharge date for admitted case-patients, 73%; and healthcare worker as occupation, 94%. Although collection of convalescent-phase sera was essential for assessing infection with SARS-CoV, samples needed for definitive laboratory determination of case status were not obtained from 46% of patients (probable case-patients: 35%; suspect case-patients: 49%; chi-square = 4.68; p = 0.03).

### Surveillance System Sensitivity and Predictive Value

Sensitivity refers to the proportion of SARS-CoV cases in the population that were detected by the surveillance system (*23*). Because SARS-CoV confirmatory laboratory testing was performed only on patients identified by the surveillance system, we cannot evaluate sensitivity for the system overall. If we limit analysis to the population of suspect and probable cases with definitive laboratory results (N = 214), we can evaluate the sensitivity of the probable case definition; all the confirmed SARS-CoV–positive patients (N = 8) had been classified as probable cases, leading to a sensitivity of 100%. The predictive value positive refers to the proportion of reported cases that actually have the health-related event under surveillance (SARS-CoV infection). The predictive value positive among cases with definitive laboratory results was 4% (8/214). The predictive value positive among the 47 probable cases with definitive laboratory results was 17%.

### Flexibility and Timeliness of Surveillance

The United States was one of many countries reporting SARS cases to WHO, which established international case definitions and reporting standards. Although flexibility was limited by the need to maintain harmonized international surveillance, U.S. surveillance remained flexible enough to incorporate frequent modifications rapidly. For example, when mainland China was added to the list of SARS-affected areas, within hours, case-patients who traveled to provinces other than Guangdong were added to the line list, and travel to mainland China quickly became the most common travel exposure (Figure 3).

The median time between symptom onset and reporting suspect or probable cases to CDC decreased during the first 12 weeks of national surveillance from 8 to 3 days. After week 12, the median time to national reporting increased to a median of 15 days, with 40% (30/76) of cases reported >50 days after illness onset. Data on date illness was reported to local and state health departments were not collected.

### Evaluation of Alternative Respiratory Pathogens

Among the 201 suspect and probable case-patients for whom serologic or PCR testing was performed at CDC, 95 (47%) demonstrated evidence of at least one alternative respiratory infection. Among specimens tested, picornavirus (enterovirus/rhinovirus) was the most common pathogen identified (29 of 114, 25%), followed by human influenza A or B virus (25/166 [15%]) and *M. pneumoniae* (22/200, 11%; Table 5). Patients with probable and suspect cases of SARS were equally likely to have an alternate cause identified (46% each). SARS-CoV–negative case-patients and those with unknown SARS-CoV status were also equally likely to have an alternate cause identified (45% and 49%, respectively). Adequate specimens were available for only two of the eight SARS-CoV–positive case-patients, one of whom also showed a fourfold or greater rise in antibodies to influenza B.

**Table 5:** Results of diagnostic testing for other infectious respiratory pathogens, U.S. SARS surveillance, March–July, 2003^a,b,c^

SARS-CoV status	*Mycoplasma pneumoniae*	*Streptococcus pneumoniae*	*Chlamydia pneumoniae* ^d^	*Legionella pneumophila*	HMPV	Influenza A or B	Parainfluenza 1, 2, or 3	RSV	Adenovirus	Picornavirus^e^	
**Positive**											
Chest imaging results^f^ positive	0/2 (0%)	0/1 (0%)	0/2 (0%)	0/2 (0%)	0/1 (0%)	1/1 (100%)	0/1 (0%)	0/1 (0%)	0/1 (0%)	--	
**Negative**											
Chest imaging results positive	3/24 (13%)	0/16 (0%)	0/24 (0%)	0/24 (0%)	2/22 (9%)	0/21 (0%)	1/22 (5%)	0/22 (0%)	0/22 (0%)	3/10 (30%)	
Chest imaging results negative	11/99 (11%)	5/71 (7%)	2/95 (2%)	0/96 (0%)	8/90 (9%)	16/84 (19%)	5/90 (6%)	2/90 (2%)	5/90 (6%)	12/45 (27%)	
**Undetermined**											
Chest imaging results positive	3/14 (21%)	1/1 (100%)	0/15 (0%)	0/14 (0%)	1/13 (8%)	1/13 (8%)	2/13 (15%)	0/13 (0%)	1/13 (8%)	4/13 (31%)	
Chest imaging results negative	5/61 (8%)	0/1 (0%)	0/61 (0%)	0/60 (0%)	1/47 (2%)	7/47 (15%)	4/47 (9%)	1/47 (2%)	3/47 (6%)	10/46 (22%)	
										
**Totals**	22/200 (11%)	6/90 (7%)	2/197 (1%)	0/196 (0%)	12/172 (7%)	25/166 (15%)	12/172 (7%)	3/172 (2%)	9/172 (5%)	29/114 (25%)	

^a^SARS, severe acute respiratory syndrome; CoV, coronavirus; HMPV, human metapneumovirus; RSV, respiratory syncytial virus; PCR, polymerase chain reaction.

^b^Denominators for results of tests vary as specimens of appropriate type and of adequate amount necessary for PCR and serologic testing were obtained only for a subset of case-patients. Positive results shown are those persons for whom evidence of acute infection was demonstrated by serologic and/or PCR testing on the specimens available for testing.

^c^Only one of the two SARS-CoV–positive case-patients had evidence of infection with another agent (influenza B). For 22 suspect and probable cases, more than one agent was identified. Combinations included: HMPV, Influenza B (FluB) + *S. pneumoniae* (N = 1); *Mycoplasma*, picornavirus + *S. pneumoniae* (N = 1); *Mycoplasma* + FluA (N = 5); HMPV + parainfluenza virus (HPIV) (N = 1); *C. pneumoniae*, adenovirus + FluB (N = 1); *Mycoplasma* + picornavirus (N = 3); adenovirus + picornavirus (N = 1); *Mycoplasma* + HPIV (N = 1); HPIV + picornavirus (N = 1); FluB + picornavirus (N = 1); adenovirus + HMPV (N = 1); HPIV + picornavirus (N = 1); HMPV + picornavirus (N = 1); *Mycoplasma* + picornavirus (N = 1); *S. pneumoniae* + picornavirus (N = 1); *S. pneumoniae* + HMPV (N = 1).

^d^All specimens tested for serologic or PCR evidence of *C. pneumoniae* were also tested for evidence of *C. psittaci*; no acute *C. psittaci* infections were diagnosed.

^e^Inclusive of rhinovirus and enterovirus.

^f^Plain film x-ray, computed tomographic scan, etc.

## Discussion

During the U.S. emergency public health response to SARS, >1,000 unexplained respiratory illnesses were reported by state and local health departments to CDC. Countless additional illnesses were investigated and rapidly ruled out for SARS by state and local health departments. Despite the large surveillance burden, discovery of the etiologic agent for SARS and development of effective diagnostic tests showed that the United States experienced limited SARS activity during the global outbreak, similar to much of Europe, Africa, Australia, and South America. There was no evidence of community transmission in the United States even though SARS-affected countries were common travel destinations for U.S. residents. Investigation of close contacts of the eight U.S. SARS-CoV–infected patients yielded one instance of secondary domestic transmission, although travel-related exposure cannot be definitively excluded for this case (*18*,*20*), and the source of exposure is considered undetermined by WHO. In addition, no healthcare workers identified by national surveillance had laboratory evidence of SARS infection, despite evidence of unprotected exposures to confirmed case-patients (*24*). While effective surveillance and timely infection-control measures likely helped limit transmission, why the United States experienced few SARS-CoV infections despite opportunities for importation and spread remains unclear.

National surveillance during the emergency response met important surveillance objectives. It identified illness clusters for further investigation, tracked progression of the epidemic in the United States, and facilitated specimen collection from suspect and probable case-patients for SARS diagnosis. This surveillance allowed for rapid and frequent updates to the healthcare and public health communities and to the public on the status of the outbreak.

Despite these successes, the system had several important limitations. Like all passive systems, it relied on astute healthcare providers to detect and report illnesses that might have been SARS. The lack of a rapid diagnostic test that could reliably diagnose SARS-CoV infection during the early phase of illness increased the workload and anxiety of clinicians, public health personnel, patients, their contacts, and the general public. Frequent, labor-intensive contact with healthcare providers was needed to obtain updated clinical information for reported case-patients. As a result, classification of patients as suspect and probable case-patients was dynamic and often changed as new information became available. This situation sometimes created seeming discrepancies between national and state and local health department case counts, which in turn complicated public communication. The evolution of the worldwide outbreak required frequent modifications of the case definition, and establishing consistent criteria to define a SARS-affected area on the basis of community transmission was difficult. Finally, the paper-based reporting system increased the difficulty of reporting to CDC and delayed timeliness of reports, and the resulting database did not allow states immediate access to their own information.

The time between disease onset and reporting to CDC increased in the latter phase of the outbreak. This increased reporting lag may reflect the growing surveillance workload as the outbreak progressed, delays in reporting until alternative diagnoses were evaluated, or a decreasing sense of urgency fueled by low disease rates and low likelihood of confirmed SARS among U.S. case-patients and lack of evidence for community transmission. The value of remaining vigilant throughout all stages of an outbreak should not be underestimated. It was critical in the context of this outbreak that infection-control measures be rapidly implemented for all suspect and probable case-patients since a single case in any area could quickly have a global impact. Evidence from Toronto, Hong Kong, Hanoi, Singapore, and Taiwan suggests that in some circumstances a single patient led to a large number of secondary cases and chains of transmission (*25*,*26*). Moreover, although most patients with SARS show radiographic evidence of pneumonia, as was observed for all the confirmed U.S. case-patients with SARS-CoV disease, in an outbreak setting, heightened vigilance and infection-control measures should be maintained for suspect as well as probable case-patients because of growing evidence that a small proportion of patients may not exhibit evidence of pneumonia and because features of pneumonia often do not develop until days 4–7 of illness (*27*,*28*). The timeliness of infection-control measures implemented for U.S. case-patients could not be assessed because relevant data were not collected as part of national surveillance.

The clinical signs and symptoms of SARS infections are similar to that of other respiratory illnesses. Empiric management of patients with respiratory illness, limited state and local capacity to perform reliable respiratory diagnostics, and lack of national surveillance for respiratory syndromes, such as pneumonia, complicated the challenge of rapid identification of SARS patients. Comprehensive testing for a variety of respiratory pathogens among patients with suspect and probable cases found that 46% had evidence of a possible infection with bacterial and viral respiratory pathogens other than SARS-CoV. Our finding that one case-patient with confirmed SARS-CoV also tested positive for influenza B infection is consistent with accumulating evidence that co-infections involving SARS-CoV and other bacterial or viral respiratory pathogens occur (*29*,*30*). This underscores the importance of obtaining convalescent-phase serum samples to make final determinations about infection with SARS-CoV and of maintaining infection-control measures despite identification of alternative agents. Moreover, in determining alternative diagnoses, the strength of the epidemiologic exposure criteria for SARS, the specificity of the diagnostic test, and the compatibility of the clinical signs and symptoms and course of illness for the alternative diagnosis should be taken into account (Tables 2 and 3). Testing for respiratory pathogens could not be completed until after the outbreak; this precluded timely re-assessment of case-patients to determine if an agent other then SARS-CoV was most likely responsible for the clinical illness. To help facilitate more timely diagnostic evaluation, CDC plans to develop real-time PCR assays for important respiratory pathogens for use by public health laboratories. Improving local capacity for diagnosing respiratory illness should strengthen national preparedness for respiratory illness threats.

In June 2003, the Council of State and Territorial Epidemiologists (CSTE) added respiratory illness due to SARS-CoV to the list of nationally reportable diseases. CDC has adopted the case definitions detailed in the CSTE position statement (*31*). This new definition, which was updated again on October 30, 2003, will improve the predictive value positive of national surveillance by considering “reports under investigation” that require monitoring and infection control as separate from cases of confirmed SARS-CoV disease that will be reported to the national system. The statement sets the stage for future SARS surveillance. CDC has developed a SARS preparedness plan for the United States that outlines in more detail recommendations for surveillance (*32*); as part of preparedness efforts, a Web-based surveillance module for SARS-CoV disease reporting is now in place.

In the absence of recognized SARS cases, initial surveillance will likely consist of sentinel case detection with a focus on unexplained illnesses in healthcare workers and travelers returning from areas that were affected by SARS in the recent global outbreak. Because hospitals experienced high rates of transmission in affected areas, infection-control teams may additionally institute passive or active surveillance for pneumonia or fevers among staff and patients, combined with diagnostic testing for SARS-CoV. The intensity of surveillance efforts will need to be tailored to the degree of local transmission within both the community and healthcare facilities. Contact tracing should rapidly identify possible early cases of secondary SARS and any unrecognized sources of infection for persons without epidemiologic links.

Challenges remain, including how best to allocate limited public health resources for preparedness planning in light of the world’s limited experience with SARS infections and how to synchronize national case definitions and reporting requirements with the systems established by international agencies, such as WHO. Although whether SARS will become a recurring problem is unclear, lessons learned while preparing for that eventuality will be important for other global infectious disease outbreaks.

## Supplementary Material

Appendix Legionella pneumophila Real-Time PCR Assay
