# Supplementary material for: SARS Surveillance during Emergency Public Health Response, United States, March–July 2003
Source: Emerg Infect Dis. 2004 Feb;10(2):185–94. doi: 10.3201/eid1002.030752 (PMC3322912; doi:10.3201/eid1002.030752)
Supplement: Appendix — Legionella pneumophila Real-Time PCR Assay [file 03-0752-app-s1.pdf]

## Appendix

### *Legionella pneumophila* Real-Time PCR Assay

Primers and a fluorescent probe were based on the nucleotide sequences of the 5S RNA gene of *Legionella pneumophila* serogroup 1 for a genus-specific assay. Primers for the 5S gene target were as follows: 5´-GCG ACT ATA GCG ITI TGG AA-3´ (forward primer) and 5´-CGA TGA CCT ACT TTC ICA TGA-3´ (reverse primer). The probe sequence was 5´-Fam-TC ACA CTA TCA TIG GCG CGG AAA QSY7-3´. They were designed by using ABI Primer Express software (Applied Biosystems, Foster City, CA). The fluorogenic probes were synthesized with a FAM (6-carboxy-fluorescein) reporter molecule attached at the 5´ end and a QSY7 NHS ester quencher dye linked to a linker arm nucleotide phosphoramidite (Glen Research, Sterling, VA) close to the 3´ end. The probes were synthesized with a 3´ terminal phosphate group to prevent extension during PCR. Primers and probes were synthesized in the Biotechnology Core Facility at CDC by using model 394-8 DNA synthesizer and standard phosphoramidite chemistries (Applied Biosystems, Foster City, CA).

Reactions were prepared in a 96-well MicroAmp optical plate (Applied Biosystems) for the 5S RNA assay by addition of a 5-µL aliquot of extracted DNA to 20 µL of a PCR master mixture consisting of 1X TaqMan 2X universal PCR master mix (Applied Biosystems), 300 nM each primer, and 100 nM fluorescent-labeled probe. Primers and probes were previously titrated to check for amplification efficiency. The amplification and detection were performed with an AB Prism 7700 Sequence Detection System (Applied Biosystems). The thermal cycle conditions were: 50°C for 2 min, 95°C for 10 min, and 40 cycles of 95°C for 15 s, and 60°C for 1 min. Standard procedures for the operation of model 7700 were followed in this study, including the use of all default program settings with the exception of reaction volume, which was changed from 50 mL to 25 mL. Cycle threshold ($C_T$) values, defined as the fraction of a cycle number at which the measured fluorescence generated by the released reporter molecule during cleavage exceeds a fixed threshold value above baseline, were automatically calculated by the instrument for each reaction. Target gene copy values were derived from a standard curve generated by plotting the $C_T$values of a dilution curve that consisted of 10-fold serial dilutions ranging from $7.5 \times 10^6$ to $7.5 \times 10^0$ copies. Each run contained at least four no-template controls to establish the baseline emission intensity of the quenched reporter dye. Negative controls (one for every five DNA extracted samples) were included. Specificity was determined by testing DNA extracted from other bacterial species that are commonly found in the human respiratory tract. Amplification plots were compared with the ones obtained by using only the serial dilutions of the *L. pneumophila* isolate as DNA templates.
